# THE NICHE CONSTRUCTION PERSPECTIVE: A CRITICAL APPRAISAL*

**DOI:** 10.1111/evo.12332

**Published:** 2014-01-26

**Authors:** Thomas C Scott-Phillips, Kevin N Laland, David M Shuker, Thomas E Dickins, Stuart A West

**Affiliations:** 1 Department of Anthropology, Durham UniversityDurham, DH1 3LE, United Kingdom; 2 E-mail: t.c.scott-phillips@durham.ac.uk; 3 School of Biology, University of St AndrewsSt Andrews, Fife KY16 9AJ, United Kingdom; 4 Department of Psychology, Middlesex UniversityLondon, United Kingdom; 5 Centre for Philosophy of Natural and Social Science, London School of EconomicsLondon, United Kingdom; 6 Department of Zoology, Oxford UniversityOxford, United Kingdom

**Keywords:** Adaptation, adaptationism, evolution, natural selection, niche, niche construction

## Abstract

Niche construction refers to the activities of organisms that bring about changes in their environments, many of which are evolutionarily and ecologically consequential. Advocates of niche construction theory (NCT) believe that standard evolutionary theory fails to recognize the full importance of niche construction, and consequently propose a novel view of evolution, in which niche construction and its legacy over time (ecological inheritance) are described as evolutionary processes, equivalent in importance to natural selection. Here, we subject NCT to critical evaluation, in the form of a collaboration between one prominent advocate of NCT, and a team of skeptics. We discuss whether niche construction is an evolutionary process, whether NCT obscures or clarifies how natural selection leads to organismal adaptation, and whether niche construction and natural selection are of equivalent explanatory importance. We also consider whether the literature that promotes NCT overstates the significance of niche construction, whether it is internally coherent, and whether it accurately portrays standard evolutionary theory. Our disagreements reflect a wider dispute within evolutionary theory over whether the neo-Darwinian synthesis is in need of reformulation, as well as different usages of some key terms (e.g., evolutionary process).

Niche construction is the process by which organisms bring about changes in their local environments, many of which are evolutionarily and ecologically consequential. In recent years a book dedicated to the topic (Odling-Smee et al. 2003), several journal special editions, and numerous journal articles have all championed the importance of niche construction for evolutionary theory (e.g., Lewontin 1982, 1983, 2000; Odling-Smee et al. 1996, 2013; Day et al. 2003; Laland and Sterelny 2006; Kendal et al. 2011; O'Brien and Laland 2012). Many interesting examples of organismal modification of environments have been described, from earthworms changing soil structure and chemistry to the effects of tree species whose roots grow in cracks in cliffs and thereby enhance the stability of mountainsides.

However, niche construction theory (NCT) is not meant to be solely a description of interesting natural phenomena. There is a further claim that the full ramifications of niche construction activity have been underappreciated, both theoretically and empirically—hence the subtitle of the book: “the neglected process in evolution” (Odling-Smee et al. 2003). This neglect is argued to result, in part, from a “major conceptual barrier” (Laland et al. 2009, p. 195) to progress within evolutionary biology, because niche construction is not widely recognized as a “fundamental cause of evolutionary change” (ibid.), equal in explanatory importance to natural selection. This perspective has been adopted or endorsed by researchers in several different areas of evolutionary biology and ecology (e.g., Kerr et al. 1999; Donohue 2005; Erwin 2008; Lehmann 2008; Krakauer et al. 2009; Post and Palkovacs 2009; Loreau 2010).

Until now, most of the commentary on niche construction has been published by its advocates. There are also a number of reviews of the book Niche Construction (e.g., Keller 2003; Abrams 2004; Ellison 2004; Hull 2004; Manning and Godfrey 2004; Vandermeer 2004; Brodie III 2005; Dickins 2005; Griffiths 2005; Krakauer 2005; Okasha 2005; Sterelny 2005), and one commentary (Dawkins 2004) that was itself a response to a commentary (Laland 2004) on the extended phenotype—an idea that is superficially similar to (but in its details and motivations very different from) niche construction. These reviews have ranged from the very positive (“With this volume, we may indeed be looking at a major breakthrough,” Vandermeer 2004, p. 474) to the very negative (“Niche construction is a phrase that should be abandoned forthwith,” Dawkins 2004, p. 381). However, none of these reviews have subjected the niche construction perspective to an in-depth critical appraisal that fully addresses the major concerns of skeptical readers.

Here we address the need for a balanced analysis of the niche construction perspective, and the literature that promotes it. We do this in the form of an adversarial collaboration, between one prominent advocate of NCT (K. N. Laland), and a team of skeptics (T. C. Scott-Phillips, D. M. Shuker, T. E. Dickins, and S. A. West). Our goal, in coming together in this way, is to provide a more evenhanded, informative, and constructive contribution to the literature than either side is likely or able to contribute on their own. Our disagreements are not about whether niche construction occurs (it clearly does) but about its implications for evolutionary theory: the advocate sees them as profound, whereas the skeptics see no reason why niche construction poses any problems for standard evolutionary theory, much less any reason for fundamental revision.

As the various issues here are to a large extent tied up with one another, we have divided the paper into several short sections and subsections, so that as much as possible the differences of opinion are clearly delineated. We begin by setting out what niche construction is, and, briefly, how NCT differs from standard evolutionary theory. We then provide a simple case study, in which both the standard account and the niche construction account of a particular example are presented alongside one another. After that, we enter into detailed discussion of the issues. Some of the examples we use to illustrate the issues are drawn from the evolutionary literature on humans. This is in part because the niche construction perspective has had a particularly significant impact in that literature, and one of the skeptics’ main motivations is to provide a counterpoint to that (see, e.g., Bickerton 2009; Fuentes 2009; Kendal et al. 2011, and articles therein; O'Brien and Laland 2012 and articles therein). However, all of the issues we discuss apply to evolutionary theory in general.

## What is Niche Construction?

Niche construction is “the process whereby organisms, through their metabolism, their activities, and their choices, modify their own and/or each other's niches” (Odling-Smee et al. 2003, p. 419). As such, niche construction is not limited to only those changes that are adaptive, those that are evolutionarily consequential, or those that impact upon the focal organism: it is any and all changes that organisms have upon the world, over any and all time frames. Homeothermic animals are surrounded by a layer of warm moist air, and this is niche construction (Lewontin 1982). Even after death niche construction continues: as a body decomposes, it will change the chemical composition of the earth around it. This extremely broad definition was adopted deliberately (Odling-Smee 1988; Odling-Smee et al. 2003), because the architects of NCT were very conscious of how seemingly trivial environmental impacts by organisms might be more important than they first appear. Good examples are the soil-generating consequences of snails that consume endolithic lichens and thereby support a desert ecosystem (Jones et al. 1997), and the seabird guano that transforms island shrub to lush grassland (Croll et al. 2005). Neither of these niche-constructing effects are adaptations (or extended phenotypes), yet both have major ecological, and plausibly evolutionary, consequences. Examples such as these that make niche construction enthusiasts reticent to define niche construction too narrowly.

Our disagreements are about the theoretical import of niche construction. Are its advocates correct to view niche construction as an evolutionary process, which changes evolutionary theory in fundamental ways? Or is it, at best, a (somewhat counterproductive) descriptive term, that refers to the effects that organisms have on environments (the skeptics’ view)?

## Standard Evolutionary Theory and NCT

In standard evolutionary theory, biological evolution is defined as change in the frequency of DNA sequences (i.e., genes and associated regulatory regions) in a population, from one generation to the next (Futuyma 2005). Evolutionary processes are generally thought of as processes by which these changes occur. Four such processes are widely recognized: natural selection (in the broad sense, to include sexual selection), genetic drift, mutation, and migration (Fisher 1930; Haldane 1932). The latter two generate variation; the first two sort it. One of these processes, natural selection, sorts this variation in such a way that, over time and on average, genes that enhance fitness are disproportionately retained at the expense of those that decrease fitness, and the result of this is adaptation (see “Niche Construction and Adaptation”). There are many factors that can cause these four evolutionary processes to occur, and for the skeptics, niche construction is one such factor. (The same is true of epigenetic effects, which change gene expression, but do not change gene frequencies; Jablonka and Raz 2009.)

NCT differs from this standard picture in several respects (see Odling-Smee et al. 2003, for a full exposition). Its central focus is not the evolution of organisms, but the coevolution of organisms and environments. It emphasizes how selection pressures are changed by evolving organisms in nonrandom, or directional, ways, stressing the role that developmental plasticity plays in generating this bias. It is this directing role that leads NCT to view niche construction as an (hitherto unrecognized) evolutionary process. NCT embraces a broadened concept of inheritance, including “ecological inheritance,” the modified environmental states that niche-constructing organisms bequeath to their descendants. Recognition of significance of extra-genetic inheritance leads the advocate to a broader than standard notion of evolution (i.e., transgenerational change in the *heritable* composition of a population). Finally, because organisms modify environments (often) in ways well suited to themselves, NCT recognizes an alternative to the standard account of how the adaptive complementarity between organisms and their environments arises.

These different views of how biological evolution occurs lie at the heart of our disagreements.

## A Comparative Example: Lactose Tolerance in Humans

To aid comparison between these views of evolution, we present an illustrative case study: two accounts of a single phenomenon, one from the standard position and one from the niche construction perspective (see “Does NCT Make Predictions or Derive New Insights that Cannot Be Made with Standard Evolutionary Theory?” for another example). We choose an example that is often presented as a flagship illustration of niche construction, and the general conceptual points that NCT seeks to make: the evolution of adult lactose tolerance in humans (e.g., Odling-Smee et al. 2003; Laland and Sterelny 2006).

### THE PHENOMENON

All human babies, but only some adults, exhibit lactose tolerance. The frequency of alleles for adult lactose absorption within a culture correlates with whether that culture has a history of dairy farming (Ulijaszek and Strickland 1993; Gerbault et al. 2011). Such data suggest that in those populations that adopted dairy farming, and hence came to rely upon dairy products throughout life, lactose tolerant alleles were selected, and/or that the spread of such alleles increased the cultural reliance on dairy products (Aoki 1986; Feldman and Cavalli-Sforza 1989; Durham 1991; Holden and Mace 1997; Tishkoff et al. 2007).

### THE STANDARD ACCOUNT

The standard neo-Darwinian account of this is as follows. The advent of dairy farming changed the local ecology. The nutritional environment had more lactose-based products than previously, and this consequently changed the selective pressures on the regulation of lactose absorption. As a result, a genetic variant that thrives in this new ecology (one for lactose tolerance) has, because of natural selection, come to increase in relative frequency within the population. In other words, the advent of dairy farming (i.e., the act of niche construction) created genetic covariance between lactose tolerance and fitness: it meant that variation in the gene(s) for lactose tolerance is now correlated with variation in fitness. Natural selection then results from this genetic covariance, that is, it changes the frequency of DNA sequences within the population.

In this respect, niche construction is no different to environmental change more generally (a change in climate, for instance). It can create genetic covariance between a phenotypic trait and fitness, and that can cause natural selection to occur. However, that change is mediated via natural selection for DNA sequences that are the most fit in this new environment (e.g., under a new climate). Environmental change is not a “process” of evolution, and, by the same logic, neither is organismic activity (see “Is Niche Construction a Distinct Evolutionary Process?”). Both are instead potential sources of the genetic covariance on which natural selection acts.

More generally, gene–environment interactions, of which the lactose case is one, are much studied with standard evolutionary theory, both presently, and before the development of NCT. One of many examples is sex-ratio distorters (male-killing bacteria; e.g., Jiggins et al. 2000; Charlat et al. 2007). In an African butterfly, *Acraea encedon*, the spread of the “male-killer” through the population led to female-biased sex ratios and a shift in the mating system of the butterfly, with females forming leks and actively soliciting copulations. Here, the spread of a male-killer gene (carried by the bacterium) changed the environment leading to behavioral shifts associated with sex-role reversal and lekking (Jiggins et al. 2000). There are a great many more such examples, all of which (in the skeptics view) serve to illustrate how niche construction activity, and gene-environment interactions more generally, have long been part of the normal activity of orthodox neo-Darwinism.

### THE NICHE CONSTRUCTION ACCOUNT

Advocates of the niche construction perspective consider the above an impoverished depiction of the causality and dynamics involved. The adoption of dairy farming is what causes this evolutionary event, and this is a manifestation of a general propensity to bias selection pressures, yet rather than niche construction being recognized as an evolutionary process it is treated as a background condition, and isolated event. Dairy farming is taken as a particularly compelling example of niche construction playing an evolutionary role because this evolutionary episode cannot adequately be characterized as caused by earlier selection.

It was research by those sympathetic to NCT and gene-culture coevolution that led to the recognition of dynamical feedback between the cultural practice and the allele for lactose persistence (i.e., the selective environment and genetic trait are coevolving; Feldman and Cavalli-Sforza 1989; Durham 1991; Holden and Mace 1997; Aoki 1986; Gerbault et al. 2011). These analyses established that dairy farming preceded genetic change. Biologists using the standard account long favored an alternative hypothesis that wrongly maintained that absorption alleles spread prior to dairy farming (e.g., Simoons 1970). The advocate sees this as one of many domains in which NCT has inspired useful research.

The advocate rejects an equivalence between niche construction and environmental change (see “The Standard Account”), arguing that there are features of niche construction that are not true of environmental change in general, and which help to explain why biological evolution takes place (or not). First, niche-construction is guided by (genetic or acquired) information, and thereby generates nonrandom environmental change, frequently driving environments into states that could not otherwise occur (Odling-Smee et al. 2003). Second, unlike environmental change stemming from independent events (e.g., climate), here ecological (even abiotic) variables are tied to rates of niche construction, often over multiple generations. NCT's population genetic models have established that the resulting dynamics are quite distinct from either cases where each trait is considered in isolation or conventional coevolution scenarios (Laland et al. 1996, 1999; Lehmann 2008; Krakauer et al. 2009; Loreau 2010); similarly, gene-culture coevolution can exhibit quite different dynamics to systems with other forms of gene–environment interaction (Boyd and Richerson 1985; Feldman and Laland 1996). Third, here the covariation between genotype and phenotype is reverse-caused and culturally contingent: evolution proceeds not because genes that cause dairy farming have higher fitness than those that do not (no such genes exist), but because dairy farming causes a change in the selective environment to favor the lactose absorption alleles, even in societies dominated by lactose intolerants.

### EVALUATIONS

As may be apparent, currently these two accounts differ more in terms of their style of explanation than dissimilarities in empirical findings or predictions. The advocate believes such dissimilarities were more manifest in the past, and that over the last two decades the standard account of the evolution of lactose intolerance has converged on that favored by NCT. In his view, NCT's emphasis on organism–environment coevolution left it particularly well placed to comprehend the evolutionary dynamics of this type of example.

From the skeptics point-of-view, the fact that an old hypothesis pursued within the standard paradigm turned out to be inaccurate and has no implications for the substantive matters at hand, because the newly established facts remain explicable within that paradigm (see “The Standard Account”). What would be necessary to justify the major claims made for NCT (see, e.g., “Inviting Misunderstanding”) would be for it to make a forward prediction of something that would *not* be explicable within the standard theory. This has not been done, and for the reasons given elsewhere in this article (especially “Does NCT Make Predictions or Derive New Insights that Cannot Be Made with Standard Evolutionary Theory?” and “Was Niche Construction Studied Before the Development of NCT”), they do not believe that it can be done.

## Niche Construction and Adaptation

### THE STANDARD APPROACH TO ADAPTATION

From the traditional perspective, the problem of adaptation is the need to explain why the fit between organism and environment is so close, in so many cases. Darwin's theory of natural selection provided a solution to this: heritable characters associated with greater reproductive success will be selected for and accumulate in natural populations. Since Darwin, there have been at least two major conceptual advances in the study of adaptation. First, the advent of population genetics united Darwin's theory with Mendelian genetics, by showing how natural selection would work via changes in gene frequency (Fisher 1930; Haldane 1932; Wright 1932; Dobzhansky 1937; Mayr 1942). Second, Hamilton (1964) showed that consequences for relatives have to be factored in to provide a more general definition of fitness (West and Gardner 2013). Of the different evolutionary processes (e.g., natural selection, genetic drift, mutation, and migration) only natural selection can explain adaptation (see “Standard Evolutionary Theory and Niche Construction Theory”).

### THE NICHE CONSTRUCTION REVISION

For NCT sympathizers, this standard account is unsatisfactory because it fails to recognize that the complementary fit between organism and environment is not simply the consequence of adaptation by natural selection, but instead of reciprocal bouts of natural selection and niche construction (“reciprocal causation”: Odling-Smee et al. 2003; Laland et al. 2011). The standard approach recognizes that organisms will be selected to change their environment in adaptive ways (e.g., Dawkins 1982), but it does not consider niche construction to be a cause of organism–environment fit (Laland 2004). Rather, it tends to focus on those aspects of niche construction that are themselves adaptations—which are, by definition, the product of selection. As advocates can envisage traits (e.g., human housing) that enhance the fit between organism and environment but are not strictly adaptations (sensu Williams 1966), they find the standard position suboptimal. This is one of the reasons why niche construction is presented as a “neglected process in evolution” (Odling-Smee et al. 2003).

### THREE (ALLEGED) PROBLEMS WITH THE NICHE CONSTRUCTION REVISION

The skeptics see a number of problems with the niche construction view of adaptation. Here we highlight three in particular.

(a) Does niche construction adapt environments to organisms in a systematic way?

The standard view is that natural selection adapts organisms to their environment in a *systematic* way, because traits that enhance fitness are disproportionately retained at the expense of those that decrease fitness (see “Standard Evolutionary Theory and Niche Construction Theory”). The skeptics see the situation with niche construction as different. Niche construction can lead to both increases and decreases in fitness, and they see no basis on which to argue that one is favored over the other (after all, niche construction is any and all changes that organisms have upon the world; see “What Is Niche Construction?”). For example, parasites sometimes compete with one another to extract as many resources as possible from hosts, and in doing so kill the host more quickly than otherwise—an obvious reduction in the host's fitness. Consequently, the skeptics see no reason to think that niche construction should act predominantly in either direction. For them, natural selection increases the fit between organism and environment in a systematic way, and hence can explain the appearance of design in nature, but niche construction does not.

NCT sympathizers wholly accept that niche construction does not always increase fitness, but they believe that niche construction will on average enhance the constructor's fitness in the short term because organisms must interact with their environments in ways that promote survival and reproductive, and random niche-constructing acts could not provide organisms with a basis for staying alive (Odling-Smee et al. 2003, ch. 4). However, complications arise because niche constructing effects can build up over time, which means that activities that are beneficial in the present may not be over multiple generations, and vice-versa (Laland et al. 1996, 1999; Lehmann 2007, 2008). This means that niche construction is more accurately characterized as favored where it increments intrinsic growth rates, or survivability, than reproductive success. Niche construction is the expression of genetic and acquired (semantic) information, information specifying how organisms should operate in their local environments to satisfy their requirements, and that information would be eradicated, by selection, or through learning, if its average effect on fitness was negative. Niche construction is a selective process, because it requires an ability on the part of organisms to discriminate, and actively sort between environmental resources, and hence to change the physical state of some factors in their environments in beneficial ways (Odling-Smee et al. 2003). Similarly, plasticity can systematically bias selection to promote or inhibit genetic differentiation, typically through differentially enhancing the organism–environment fit (West-Eberhard 2003; Pfennig et al. 2010; Fitzpatrick 2012). These processes generate a systematic and directional bias to niche construction.

The skeptics do not see why any of these points mean that niche construction adapts environments to organisms in a systematic way. Yes, niche construction may be based upon adaptive plasticity, but that plasticity is already well-studied and understood with standard evolutionary theory. Furthermore, the niche construction of any given organism has, as advocates point out, a multitude of effects on other organisms and species, and in those cases the arguments above would not seem to apply anyway.

In the end, whether niche construction does in fact adapt environments to organisms in a systematic way is, potentially, an empirically tractable issue, although we disagree on how easily this could be demonstrated or quantified.

(b) When explaining adaptation, is niche construction equivalent to natural selection?

Advocates consider (internally and externally expressed) constructive processes, such as niche construction, to be of the same explanatory importance as natural selection: “Niche construction should be regarded, after natural selection, as a second major participant in evolution” (Odling-Smee et al. 2003, p. 2). The skeptics dispute this on two grounds. First, as described above, the skeptics argue that this claim conflates evolutionary processes with the causes of those processes. Niche construction, like all environmental change, can cause evolutionary processes to occur, but this does not make it an evolutionary process itself (see “Standard Evolutionary Theory and Niche Construction Theory”; also “Is Niche Construction a Distinct Evolutionary Process?”). Thus, it is, for the skeptics, a category error to conflate the causes of evolutionary change with evolutionary change itself. Second, the skeptics maintain that niche construction does not act in a systematic way, whereas natural selection does.

With regards the first point, the advocate believes that data amassed by the NCT and developmental bias literatures leaves the skeptics’ stance outdated, and that there has become a pressing need to recognize that, through systematically biasing selection, developmental processes carry some of the responsibility for the direction and rate of evolutionary change (Gould 2002; Arthur 2004; Laland et al. 2011). Concerning the second point, the advocate rejects the suggestion that niche construction does not act in a systematic way (see “Does Niche Construction Adapt Environments to Organisms in a Systematic Way?”). For instance, if animals did not behave in a systematic way there could be no science of animal behavior. NCT advocates see little virtue in the skeptics’ stance, which they regard as too simple.

c. Does the niche construction perspective make an unambiguous, general prediction about adaptation?

The third problem that the skeptics see with the niche construction approach to adaptation is that, unlike the standard approach, it does not make an unambiguous and clear prediction about the natural world.

Standard evolutionary theory predicts that natural selection will lead to organisms that appear as if they have been designed to maximize their (inclusive) fitness subject to the normal constraints of genetic architecture, history, trade-offs, and so on (see “The Standard Approach to Adaptation”). This result is the cornerstone for modern evolutionary biology, and it has had vast empirical success (Westneat and Fox 2010; Davies et al. 2012). For the skeptics, niche construction does not support a similarly unambiguous, general prediction—because niche construction activity can lead to longer term increases or decreases in fitness, with no process by which to filter these. Consequently, the skeptics think that it is misleading to consider natural selection and niche construction as equal and equivalent processes, and that to do so potentially spreads confusion about what each represents. Put simply, natural selection has a property that it leads to the maximization of (inclusive fitness; West and Gardner 2013), but niche construction does not.

The advocate acknowledges that NCT does not currently make any general formal statement concerning adaptation along these lines, although he does not rule out the possibility that the claim that niche construction is typically adaptive could be formalized in the future. However, he views this “concern” as misplaced, as the claim that niche construction, and developmental processes in general, are as explanatorily important as natural selection is based on the argument that developmental processes channel selection along particular pathways (Gould 2002; West-Eberhard 2003; Brakefield 2006; Müller 2007), not that they function like selection. He views niche construction as operating like Gould's (2002) “active constraints” to impose directionality on evolution by biasing the action of selection.

## Skeptical Questions about NCT

In this section, we pose, and then answer, a series of skeptical questions about the substance of NCT.

### DOES NCT MAKE PREDICTIONS OR DERIVE NEW INSIGHTS THAT CANNOT BE MADE WITH STANDARD EVOLUTIONARY THEORY?

We all agree that it is not logically necessary to use NCT to study and make scientific predictions about the natural world. NCT does *not* suggest: (i) that standard neo-Darwinism fails to recognize that organisms modify environmental states; (ii) that standard neo-Darwinism only considers selection pressures that emanate from the abiotic environment; or (iii) that it is not possible, even in principle, to provide explanatory accounts of biological phenomena with standard neo-Darwinism. In the light of this agreement, the skeptics see no reason to think that whatever predictions and insights NCT leads to, the same predictions could not be derived from standard evolutionary theory.

The advocate agrees with the spirit of this comment, in the sense that the standard approach can be used to investigate any aspect of the biological world, but anticipates that the two frameworks will often lead to different predictions, and he maintains that standard neo-Darwinism does not provide *satisfactory* explanations for some phenomena. Furthermore, for the advocate the issue is not whether the same insights can be derived from the conventional perspective, but whether they actually are. Conceptual frameworks channel thinking, and the advocate believes some findings have followed more easily from the niche-construction perspective, which in itself illustrates its utility.

Consider, for illustration, the beaver's dam. From a conventional perspective, beaver's dams are “extended phenotypes” (Dawkins 1982), which evolve in essentially the same way as other aspects of the beaver phenotype—through the differential selection of dam-building alleles. In the advocates view, this implies that existing theoretical models adequately describe their evolution, and as a consequence the extended phenotype stimulates little new population genetic theory (the skeptics do not agree). In contrast, NCT emphasizes how beavers are keystone species, whose activities dramatically alter local environments, modifying nutrient cycling and decomposition dynamics, shaping ecological community composition and diversity, and thereby inadvertently modifying selection on virtually all aspects of the beaver phenotype. Moreover, such modified selection pressures remain as long as the dam, lake, and lodge remain and, as these are maintained by families of beavers over generations (Naiman et al. 1988), that can be longer than the lifetime of an individual beaver. NCT has drawn attention to the hitherto largely neglected feedback from the legacy of modified selection (ecological inheritance), as it has to other forms of niche construction that do not fit the extended phenotype model (e.g., constructions that result from by-products, acquired characters or multiple species activities). Because it emphasizes how the effects of niche construction can build up over time, NCT was well placed to observe how ecological inheritance affects the evolution of, often distant, descendants. NCT models of ecological inheritance, inspired by examples like the beaver, have shed light on the evolutionary dynamics of such cases, for instance, revealing timelags in the response to selection as environmental resources accrue/deplete, with associated momentum and inertia effects (Laland et al. 1996, 1999), autocatalytic effects in which (even selectively disadvantageous) niche-constructing traits can drive themselves to fixation through hitchhiking on other traits favored in constructed environments (Silver and di Paolo 2006), and associated impacts on cooperation and dispersal, where otherwise disadvantageous niche-constructing traits are favored because of the benefits that accrue to distant descendants, or are selected solely in constructed habitat (Lehmann 2007, 2008; Kylafis and Loreau 2008; van Dyken and Wade 2012). For the advocate, this is another domain in which NCT has proven productive.

The skeptics happily acknowledge that it is possible that the niche construction perspective has brought some previous unstudied or understudied topics to the foreground, of which the beaver dam may be a good example. However, they see no reason to think that such topics could not or would never have been studied with the conventional framework (there will always be interesting but unstudied or understudied topics available for research). The beaver dams are much like the advent of dairy farming (see “A Comparative Example: Lactose Tolerance in Humans”): an instance of environmental change, which has changed selective environments. The research cited earlier describes the effects that these changes have. In short, the skeptics entirely agree that it is important to acknowledge the evolutionary consequences of niche construction activity (which standard theory does, and has ample methods with which to such these consequences), but they disagree that this acknowledgement has the profound consequences for evolutionary theory claimed for it.

### WAS NICHE CONSTRUCTION STUDIED BEFORE THE DEVELOPMENT OF NCT?

We all agree that aspects of niche construction have been intensively and productively investigated, for many decades, without recourse to NCT. The consequences of organismal activity on the selective environment have been part of evolutionary theory since Darwin's writings on earthworms and corals (1851, 1881), and featured in the earliest mathematical models of natural selection (Fisher 1930; Haldane 1932). Since then, a diverse theoretical and empirical literature has developed within both evolutionary biology and evolutionary ecology using the neo-Darwinian framework to examine specific traits where organisms modify the world around them (Westneat and Fox 2010; Davies et al. 2012). Three specific examples are: (i) social evolution theory, which examines how the behavior of one organism affects the selective environment of another (Hamilton 1964; Frank 1998); (ii) coevolutionary theory, which rests entirely on the fact that organisms often modify the world such that they change the selection pressures that act upon others, often in ways that feed back to affect selection on themselves (Thompson 1994); and (iii) sexual selection, which is driven by interactions, such as how female choice determines selection on male ornaments (Darwin 1859; Andersson 1994).

Research in these areas has led to the development of numerous theoretical methods that apply to niche construction scenarios, such as how to deal with class-structured populations, reproductive value, the state of the organism, direct and indirect effects, and simultaneous changes at many genetic loci (Mangel and Clark 1988; Taylor 1990, 1996; Barton and Turelli 1991; Taylor and Frank 1996; Moore et al. 1997; Frank 1998; Wolf et al. 1999; Kirkpatrick et al. 2002; Grafen 2007; Gardner et al. 2007). Furthermore, the ways that environments change over time can be, and often are, taken into account in evolutionary analysis. For example, ecologists explicitly acknowledge that environments can change or be consistent across generations with notions such as ecological succession and facilitation (e.g., Begon et al. 2006).

In short, numerous forms of niche construction have been studied extensively and successfully before the advent of NCT, and independently of it since its advent (a point often made in the niche construction literature itself, e.g., Odling-Smee et al. 2003). Nevertheless, the niche construction literature claims that the import of niche construction is neglected by evolutionary biology (e.g., “… not well described or well understood …,” Odling-Smee et al. 2003, p. 1). The skeptics read this as a contradiction and denial of the successes of the standard approach to niche construction activity, surveyed above.

The advocate reiterates that the claim is not that niche construction has never been studied (much of the aforementioned theory is reviewed in Odling-Smee et al. 2003, monograph, ch. 2), but rather that niche construction had hitherto been investigated in a piecemeal manner, without any comprehensive general investigation of its full evolutionary ramifications, leading to the neglect of important phenomena, and without treating niche construction as a process. He also notes that Lewontin's (1982, 1983) and Odling-Smee et al.'s (1996, 2003; Odling-Smee 1988) original focus was on ecological niches, for instance, on indirect forms of evolutionary and ecological feedback to the constructor, and indirect forms of connectance in ecosystems. In contrast, topics such as sexual selection and social evolution, which are concerned with direct (i.e., social) interactions between biota, were viewed as only tangentially relevant. Subsequently, NCT broadened out to encompass the social niche, but the primary focus remains indirect interactions mediated by physical resources. The advocate points out that the view that key aspects of niche construction have been neglected is echoed by researchers working outside of NCT, including ecologists studying ecosystem engineering (e.g., Jones et al. 1997) and eco-evolutionary dynamics (e.g., Post and Palkovacs 2009), and evolutionary biologists studying the role of developmental plasticity in evolution (e.g., West-Eberhard 2003).

We disagree over whether this nuance is sufficiently clear in the niche construction literature, but we all agree that any claim that niche construction activity was simply not studied prior to the advent of NCT would: (i) obscure links between areas of research, in a way that can impede progress and spread confusion; (ii) underplay the past advances in areas that could be termed niche construction; and (iii) potentially alienate researchers from fields such as evolutionary genetics or behavioral and evolutionary ecology who would say they have been doing this all along.

### WHAT IS THE SCOPE OF NICHE CONSTRUCTION?

As aspects of niche construction have been successfully studied without using the term, does the niche construction perspective suggest that these areas should be redefined, or reconceptualized, as niche construction? The advocate's response is to appeal to pragmatism: where it is useful to do so, we should label phenotypic outputs as niche construction, but if it is not useful, there is no need. On this view, the correct question to ask is “When is it useful to emphasize that organisms engage in niche construction, and when is it not?,” and this utility is to be judged in terms of the insights generated. For instance, whether, say, discarded shells or hoof prints need to be treated as an instance of niche construction depends on whether the shells or indented soils provide resources for other organisms, and/or accrue in space and time to affect selection pressures on descendant populations. More generally, we agree that where theory and methods already exist for studying a particular phenomenon (e.g., sexual selection; social evolution), it is only useful to use a niche construction perspective if doing so generates new insights (examples of NCT-sympathetic treatments of sexual selection are Oh and Badyaev 2010; Cornwallis and Uller 2010).

The skeptics among us agree that pragmatism is an important part of scientific thinking, and that it is frequently useful to look at things from multiple perspectives (Maynard Smith 1983). At the same time, they find it difficult to reconcile this approach with claims that niche construction is “more accurate” than the standard theory, that the evolutionary ramifications of niche construction have “not been subject to a great deal of investigation,” and that evolutionary theory contains within itself a “major conceptual barrier” (Odling-Smee et al. 2003; Laland et al. 2009). It is not readily apparent to the skeptics how one can simultaneously call for a “reformulation” or “overhaul” of evolutionary theory, and at the same time acknowledge that many of the fields that study the effects that organisms have upon the world around them have made good progress without niche construction (see also Keller 2003; Dawkins 2004). Nonetheless, the advocate maintains that this position is tenable: he believes NCT brings with it a useful difference in focus from standard thinking, which has led to new insights and findings, and contributes to the satisfactory integration of evolutionary theory with adjacent disciplines, including developmental biology, ecology, and the human sciences (Laland et al. 2013; Odling-Smee et al. 2013). The skeptics agree with the importance of integration with other disciplines but, for the reasons listed elsewhere in this paper, do not think that NCT aids this goal.

### IS NICHE CONSTRUCTION A DISTINCT EVOLUTIONARY PROCESS?

NCT argues that niche construction is a distinct evolutionary process, potentially of equal importance to natural selection. The skeptics dispute this. For them, evolutionary processes are processes that change gene frequencies, of which they identify four (natural selection, genetic drift, mutation, migration; see “Standard Evolutionary Theory and Niche Construction Theory”). They do not see how niche construction either generates or sorts genetic variation independently of these other processes, or how it changes gene frequencies in any other way. For instance, although organismic behavior can bias the distribution of variants subject to selection, that genetic variation is mutational in origin. Hence, the skeptics argue, niche construction is not an evolutionary process, in and of itself, and claims to the contrary conflate *causes* of change (niche construction, environmental change, etc.) with the evolutionary *processes* by which those changes occur (natural selection, genetic drift, mutation, migration). This seems to them both unnecessary and, more importantly, unhelpful.

In contrast, NCT adopts a broader notion of an evolutionary process, one that it shares with some other evolutionary biologists (e.g., Endler 1986). Although the advocate agrees that there is a useful distinction to be made between processes that modify gene frequencies directly, and factors that play different roles in evolution, he also believes that there are now sufficient data to warrant a rethink, and to recognize as evolutionary processes a new category of phenomena that systematically bias the action of selection, which includes niche construction, but also “developmental bias” (Arthur 2004; Müller 2007). For the advocate, a failure to recognize these factors as evolutionary processes leads to an inaccurate and impoverished account of evolutionary dynamics. He views his stance to be intellectually aligned with similar calls to recognize developmental processes (e.g., developmental plasticity) as playing evolutionary roles (West-Eberhard 2003; Pfennig et al. 2010; Fitzpatrick 2012).

### HAVING IT BOTH WAYS?

To the skeptics, the niche construction literature appears to want to have things both ways. On the one hand, niche construction is defined in the broadest possible sense, as anything and everything an organism does to its environment—which is everything it does at all (since all activity must have some impact on the physical world; see “What is Niche Construction?”). On the other hand, the niche construction literature claims that niche construction has not been subject to much investigation by evolutionary biology. For example, the first page of the book *Niche Construction* states that: “This… [niche-constructing] role for phenotypes in evolution is not well described or well understood by evolutionary biologists and *has not been subject to a great deal of investigation*” (Odling-Smee et al. 2003, p. 1, italics added). This juxtaposition, between a definition of niche construction that includes everything an organism ever does, and the claim that it has not previously been studied by evolutionary biology, seems to them inconsistent.

For the reasons given previously, the advocate maintains that a broad characterization of niche construction is more useful than a narrower one. Although it is a truism that all organisms modify their environments, it is far from a truism that these activities are ecologically or evolutionarily consequential. He sees no inconsistency between this broad definition of niche construction and NCT's radical stance because, in his view, scientific theories must be judged on their usefulness and in his opinion the growth of interest in niche construction is testament to its utility. The advocate maintains that (outside of restricted domains) the role that niche construction plays as an evolutionary process that directs selection by changing the *ecological* environment remains under-investigated and poorly understood. More generally, he believes the roles that developmental processes (which includes niche construction) play in evolution remain massively underappreciated, that NCT helps draw attention to these, and that NCT is part of a broader movement seeking change in how evolutionary biology is characterized (i.e., working towards an Extended Evolutionary Synthesis).

### INVITING MISUNDERSTANDING?

The skeptics believe that prominent claims made to promote NCT overstate its importance and invite misunderstanding. Here are three examples (italics added in all cases): “[neo-Darwinism] fails to recognize a fundamental cause of evolutionary change … niche construction” (Laland et al. 2009, p. 195); “The changes to the evolutionary process brought about by niche construction … are sufficiently important and occur sufficiently frequently to warrant an overhaul in evolutionary thinking.” (Day et al. 2003, p. 82); “NCT differs from standard evolutionary theory… in recognizing that the evolution of organisms is co-directed by both natural selection and niche construction” (Kendal et al. 2011, p. 785). For the skeptics, one reasonable interpretation of these claims, and many others like them, is that standard evolutionary theory does not and perhaps cannot take into account the effects that organisms have upon the world. More explicitly: that it is not possible to study (at least some of) the effects that organisms have upon the world with standard evolutionary theory, even in principle—and this is why we need NCT. Certainly, one published review of the book Niche Construction explicitly read it this way: “Natural selection is depicted as resulting [only] from inanimate and abiotic features of the environment” (Brodie III 2005, p. 249).

The advocate believes that this interpretation is a distortion, that most readers have not read the niche construction literature in this way, and that such a reading lacks credibility because it is so manifestly false to anyone with the most basic knowledge of evolutionary biology and ecology. It is certainly not the meaning intended by the architects of NCT. Rather, such quotations should be read as implying that standard evolutionary theory does not recognize niche construction, and its legacy over time (“ecological inheritance”), to be distinct evolutionary processes, and that as a result important phenomena have been underinvestigated. Although this might seem like a subtle change of emphasis, it brings with it a suite of consequences, discussed in previous sections, that to advocates are substantial enough to justify the language deployed. This is in line with statements from other radical elements within evolutionary biology (e.g., “These facts call for a fundamental revision of ideas about the origins of organic diversity,” West-Eberhard 1986, p. 1391; developmental bias is “an equal partner [to natural selection] rather than a bit-part player,” Arthur 2004, p. 193). The skeptics find it hard to reconcile this agenda of a “subtle change of emphasis” with the revolutionary tone they perceive in statements such as “fails to recognize,” “major conceptual barrier,” and “reformulation of evolutionary theory.”

## Summary

Many previous articles have promoted the utility of the niche construction perspective. Here we have subjected that literature to a critical appraisal. We recognize that our presentation, which juxtaposes two positions, is somewhat polarized, and that researchers may accept some aspects of NCT without necessarily accepting others (e.g., Bonduriansky 2012). Nonetheless, we trust that the exchange is useful in highlighting the main issues at stake, which are summarized in table1.

**Table 1:** Comparison of standard evolutionary theory and niche construction theory (NCT)

Question	Skeptics	Advocate
Can standard evolutionary theory be used to study niche construction?	Yes	Yes
Was niche construction studied before the advent of NCT?	Yes	Yes
Were important aspects of niche construction understudied prior to NCT?	No	Yes
Does NCT theory make any predictions that could not, in principle, be made with standard theory?	No	No, but many useful theories (e.g., kin selection) make predictions that could have been made by preexisting theory.
Has NCT made predictions that were not made by standard theory? Has NCT generated novel empirical and theoretical insights?	Yes, but any new approach could do this, by simply picking an unstudied area and developing a model.	Yes
Can the evolution of traits such as lactose tolerance be explained by standard evolutionary theory?	Yes	Yes, but in an impoverished way.
Is niche construction a distinct evolutionary process?	No. Evolutionary change is change in gene frequencies, and evolutionary processes are those processes that bring about this. There are four such processes: natural selection, mutation, migration and drift. It is a category error to add niche construction to this list.	Yes. Developmental processes that systematically bias the action of selection merit recognition as evolutionary processes.
Is natural selection the only process that can explain the pervasive adaptive complementarity of organism and (biological) environment?	Yes	No. Organism–environment complementarity also results from niche construction (and other developmental processes).
Does niche construction adapt environments to organisms in a systematic way?	No. Niche construction can lead to changes in the environment that either increase or decrease organism fitness.	Yes. Niche construction must typically (although not inevitably) be adaptive for the constructor, at least in the short term.
Is niche construction of equivalent explanatory importance to natural selection?	No. Only natural selection leads to the appearance of organismal design.	Yes. Niche construction (and other developmental processes) systematically bias the direction and rate of selection.
Does niche construction make a general, formal prediction about organismal design?	No	Not yet, but this is feasible.
Does the neo-Darwinian modern synthesis need to be reformulated as part of an Extended Evolutionary Synthesis?	No	Yes

At the heart of this exchange lie differences in perspective germane to several current evolutionary debates. To illustrate, we here highlight two. The first is the question of what counts as an evolutionary process. The skeptics probably represent the majority position: evolutionary processes are those that change gene frequencies. Advocates of NCT, in contrast, are part of a sizable minority of evolutionary biologists that conceive of evolutionary processes more broadly, as anything that systematically biases the direction or rate of evolution, a criterion that they (but not the skeptics) feel niche construction meets. The second difference concerns the merits of the adaptationist stance. The skeptics among us embrace adaptationism, see natural selection as the ultimate source of organism-environment fit, have a gene-centered view of evolution, and view nongenetic inheritance as a proximate mechanism rather than a distinct evolutionary process. NCT enthusiasts, in contrast, influenced by Lewontin and Gould's writings, are frequently sympathetic to a structuralist tradition that stems from developmental biology (e.g., Waddington 1959), which emphasizes not only constraints on adaptation but also the evolutionary significance of processes other than selection, and a broader notion of inheritance.

These specific differences reflect a broader difference of opinion within evolutionary biology, between those happy with the standard neo-Darwinian synthesis, and those that are dissatisfied with it in one way or another. The skeptics among us are part of the former group, the advocate part of the latter. Some of the other places in which this split can be observed are the various debates about: the role of epigenetic (and other forms of extra-genetic) inheritance in evolution; the role of developmental plasticity and developmental constraints in evolution; and the utility of the distinction between ultimate and proximate explanations (see, e.g., West-Eberhard 2003; de Jong 2005; Jablonka and Lamb 2005; Laland et al. 2011; Dickins and Barton 2012; Dickins and Rahman 2012; Danchin 2013).
